# Direct Infection of Dendritic Cells during Chronic Viral Infection Suppresses Antiviral T Cell Proliferation and Induces IL-10 Expression in CD4 T Cells

**DOI:** 10.1371/journal.pone.0090855

**Published:** 2014-03-10

**Authors:** Carmen Baca Jones, Christophe Filippi, Sowbarnika Sachithanantham, Teresa Rodriguez-Calvo, Katrin Ehrhardt, Matthias von Herrath

**Affiliations:** Type 1 Diabetes Center, Developmental Immunology, La Jolla Institute for Allergy and Immunology, La Jolla, California, United States of America; University of Iowa, United States of America

## Abstract

Elevated levels of systemic IL-10 have been associated with several chronic viral infections, including HCV, EBV, HCMV and LCMV. In the chronic LCMV infection model, both elevated IL-10 and enhanced infection of dendritic cells (DCs) are important for viral persistence. This report highlights the relationship between enhanced viral tropism for DCs and the induction of IL-10 in CD4 T cells, which we identify as the most frequent IL-10-expressing cell type in chronic LCMV infection. Here we report that infected CD8α^neg^ DCs express elevated IL-10, induce IL-10 expression in LCMV specific CD4 T cells, and suppress LCMV-specific T cell proliferation. DCs exposed *in vivo* to persistent LCMV retain the capacity to stimulate CD4 T cell proliferation but induce IL-10 production by both polyclonal and LCMV-specific CD4 T cells. Our study delineates the unique effects of direct infection versus viral exposure on DCs. Collectively these data point to enhanced infection of DCs as a key trigger of the IL-10 induction cascade resulting in maintenance of elevated IL-10 expression in CD4 T cells and inhibition of LCMV-specific CD4 and CD8 T cell proliferation.

## Introduction

The host-pathogen relationship in chronic viral infections requires the establishment of equilibrium between the host immune response and viral replication. While this balance of competing interests aids in protecting the host from the immunopathologic consequences of continuous inflammation, such a truce can also result in the prolonged persistence of the virus within its host. Studies over the last decade have identified several characteristics common to multiple persistent viral infections including elevated levels of systemic IL-10 and T cell exhaustion [1]–[8].

IL-10, a pleiotropic cytokine produced by a variety of immune cells including both adaptive and innate effectors, acts as a regulator of Th1 and Th2 responses, aiding in the contraction phase of a normal Th1 immune response. In addition to its role as a negative regulator, IL-10 also supports the development of B cell responses, and regulatory T cell development and function [9]. Enhanced dendritic cell (DC) infection, elevated IL-10 expression and rapid T cell exhaustion (a state of diminished effector function, increased inhibitory receptor expression and altered transcriptional profiles), are hallmarks of chronic, but not acute, lymphocytic choriomeningitis (LCMV) infection [3], [5], [10]–[16].

The LCMV model of acute versus chronic viral infection employs a naturally arising mutant strain, Clone 13 (Cl13), in comparison with the parental strain, Armstrong 53b (Arm). Infection of mice with Cl13 provides an elegant model of chronic viral infection, whereby five nucleotide mutations, resulting in only three amino acid substitutions in the viral sequence, have profound effects on the outcome of infection. These small genomic changes translate to discreet differences in viral tropism (enhanced infection of DCs and fibroblastic reticular cells) and subversion of the immune response (elevated IL-10 expression and early T cell exhaustion) [14], [16]–[20]. The LCMV model is unique among chronic viral infection models in that the viral and host factors contributing to either acute viral infection and rapid clearance, or persistent viral infection, can be studied using nearly identical viruses with dramatic differences in the hosts’ ability to control infection.

We and others have shown that IL-10 receptor blockade can resolve chronic LCMV infection; however, the underlying dynamics of elevated IL-10 production remain poorly understood [3], [5]. Notably, it has remained unclear which cell types prime IL-10 production in chronically infected hosts *in vivo* and whether elevated IL-10 expression is a consequence of enhanced viral tropism for DCs.

Understanding the dynamics of IL-10 induction and the role infection of DCs may play in promoting chronic LCMV infection has been a highly active area of research as it may have significant implications in a variety of clinically relevant viral infections. As such, several groups have taken different approaches to unveil the critical factors contributing to elevated IL-10, some with seemingly conflicting results. Wilson *et al.* identified macrophages as the largest number of IL-10-expressing cells in Cl13 infection, and their secretion of IL-10 as the dominant factor in the suppression of LCMV-specific CD4 T cell proliferation *in vitro* [21]. However the Oldstone group did not find macrophages to be a significant source of IL-10, and rather concluded that infected CD8α^neg^ DCs contribute the majority of IL-10 in the serum [22].

In the current study, we sought to more clearly define the role that enhanced viral tropism for DCs plays in the induction and maintenance of elevated IL-10 and T cell exhaustion during chronic LCMV infection. We identified functional differences specific to infected CD8α^neg^ DCs that contribute to the suppression of CD8 T and CD4 T cell proliferation and the induction of IL-10 in naïve cells. Infected DCs both expressed elevated IL-10 and were able to promote IL-10 expression in CD4 T cells, which constituted the largest population of IL-10-expressing cells in Cl13 infection at all time points observed.

## Results

### CD4 T cells are the most frequent IL-10-expressing cell type in chronic LCMV infection

**Figure 1:**
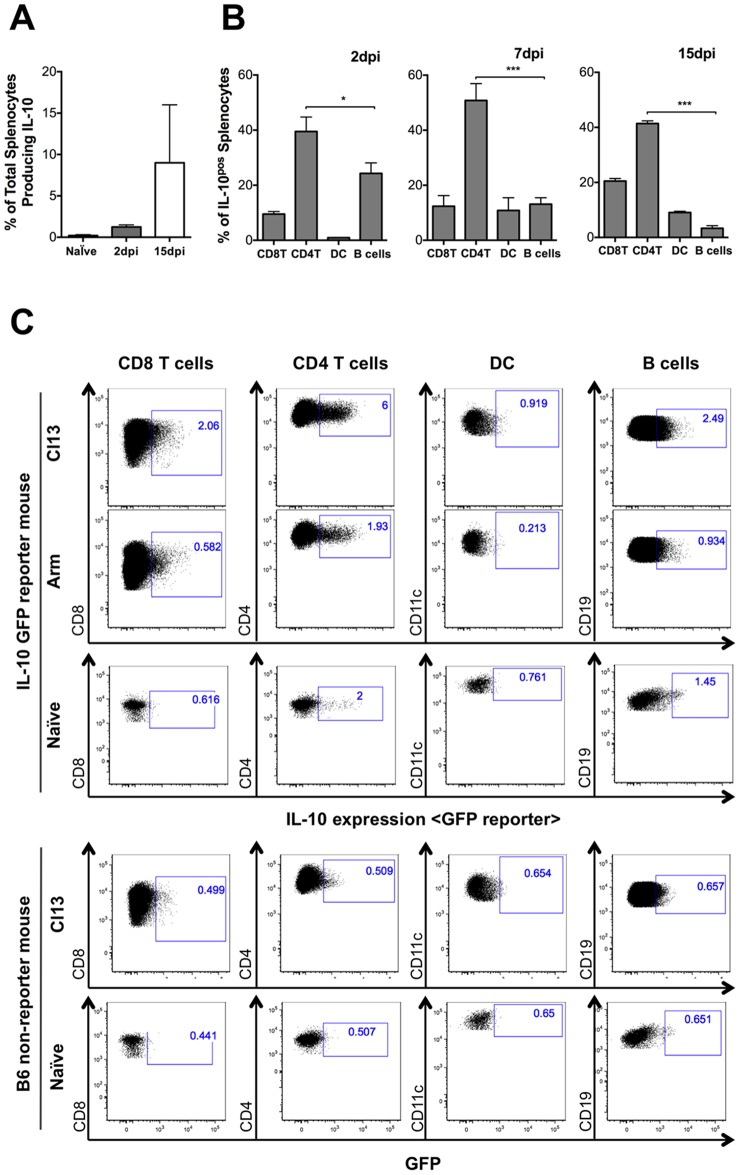
CD4 T cells are the most frequent IL-10 expressing cell type in chronic LCMV infection. Adult IL10-GFP reporter mice were infected with Cl13 and spleens harvested at the indicated days post-infection. (A) The frequency of IL-10 positive cells, as measured by IL-10 GFP reporter activity, in total splenocytes is shown. (B) Splenocytes from IL-10 GFP reporter mice were stained for CD11c, CD90, B220, CD4 and CD8 at the indicated times post-infection. Shown is the frequency of IL10^pos^ splenocytes (as determined by GFP reporter expression) that stain positively for CD4 T cell, CD8 T cell, B cell and DC markers. (C) IL-10 GFP reporter mice (top three rows) or wildtype C57BL/6 non-reporter mice (bottom two rows) were infected (as indicated) and spleens harvested 15 days post infection. Dot plots depict unstimulated IL-10 GFP reporter expression directly *ex vivo*. Naïve IL-10 GFP reporter mice indicate baseline IL-10 expression. Naïve and Cl13 infected wildtype C57BL/6 are used as autofluorescence gating controls. (A and B) n = 6 for Cl13 infected mice and n = 5 for uninfected. (C) n = 4 for Cl13, 3 for Arm, 2 naïve and 2 Cl13 B6 nonreporter gating controls. Data are representative of one of three (A) or four (B) independent experiments. Standard Deviation is shown.

In order to examine the role that enhanced DC tropism might play in the induction and maintenance of persistent LCMV infection, including contribution to IL-10 levels, we utilized an IL-10 reporter mouse strain expressing GFP under the control of the IL-10 promoter on a C57BL/6 background. These IL-10-GFP (“TIGER”) mice have an internal ribosome entry site (IRES) green fluorescent protein expression cassette inserted upstream of the polyadenylation site in the IL-10 gene [23]. We infected IL-10-GFP mice with LCMV Cl13 or Arm and monitored GFP/IL-10 expression in total splenocytes following infection (Fig. 1A). GFP/IL-10 expression was readily detected as early as 2 days post-infection (dpi) in Cl13-infected mice, and by 15 dpi we observed a ten-fold increase (over naïve reporter mice) in the proportion of cells expressing IL-10 (Fig. 1A). GFP/IL-10 induction was below detection limits in total splenocytes from Arm-infected reporter mice (data not shown).

While multiple cell types were found to produce IL-10 *in vivo*, including CD8 T cells, B cells and DCs, at all time points CD4 T cells were the single largest group of IL-10-expressing cells, comprising 40–50% of all IL-10^pos^ splenocytes (Fig. 1B). GFP/IL-10 expression levels in CD8 T, CD4 T, DC and B cells 15 dpi, also point to CD4 T cells as key IL-10 producing cell types, with the largest proportion of cells expressing IL-10 (6% of CD4 T cells as compared to 2% of CD8 T cells) and the highest GFP/IL-10 MFI among the IL-10^pos^ cells (Fig. 1C). While less than 2% of CD4 T cells from Arm infected mice were found to express IL-10, the frequency of cells which were IL-10^pos^ were equivalent to IL-10 expression observed in naïve animals, indicating no induction over baseline IL-10 expression in CD4 T from Arm infected mice.

### The majority of IL-10 expressing splenocytes are uninfected

**Figure 2:**
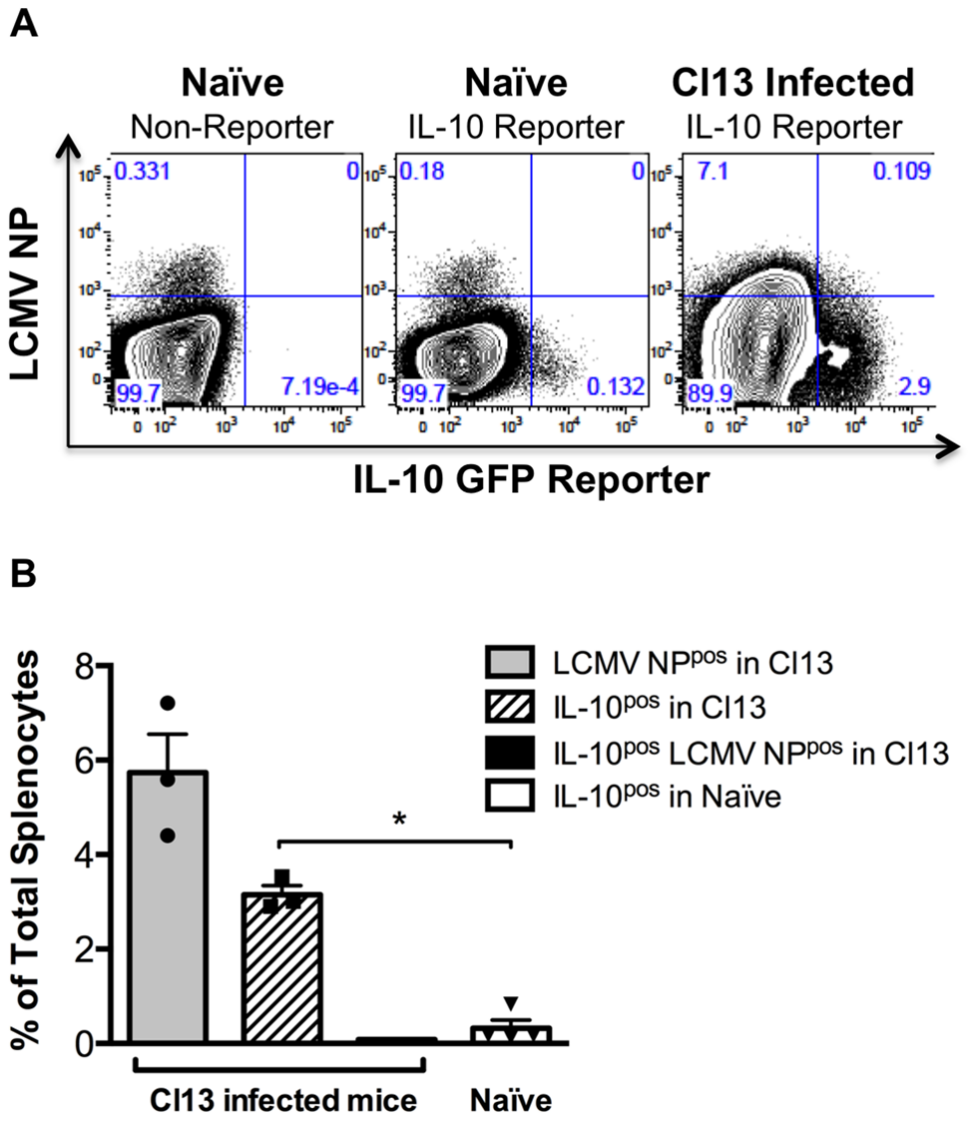
The majority of IL-10 expressing splenocytes are uninfected. Adult IL-10 GFP reporter mice were infected with Cl13 and spleens harvested 15 days post infection. Splenocytes were stained with α-LCMV NP Ab. (A) Representative FACS plots are displayed. (B) The frequency of splenocytes that were LCMV NP^pos^ (grey bar), IL-10 (GFP^pos^) (hatched bar), double positive (black bar) and the frequency of splenocytes that are IL-10^pos^ in naïve reporter mice (white bar) is shown. n = 3 Cl13 and n = 4 for uninfected. SEM shown.

**Figure 3:**
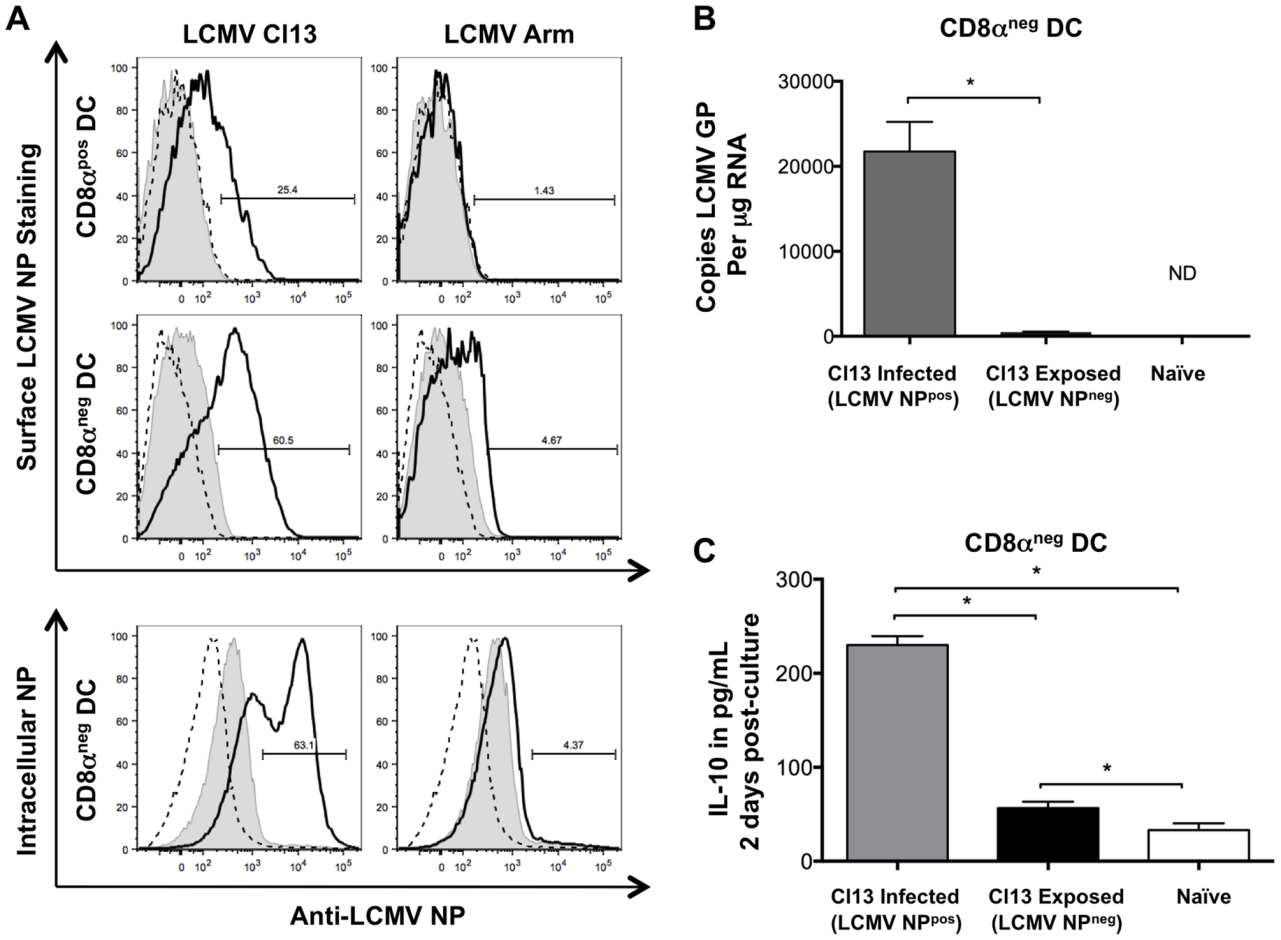
Clone 13 efficiently infects CD8α^neg^ CD11c^pos^DC. Adult C57BL/6 were infected with LCMV Arm, or Cl13 and spleens harvested 7 days post infection. CD11c^pos^ cells were positively selected by MACS bead isolation following CD19/90 depletion. (A) Isolated cells were stained for CD11c, CD90, CD19, CD8α and cell surface (top two rows) LCMV NP or intracellular (bottom row) LCMV NP. Gating controls are as follows: Naïve DC (dashed line), DCs from Cl13 infected mice incubated with isotype control antibody for LCMV NP Ab (filled grey histogram). (B & C) CD8α^neg^ DCs were sorted based on cell surface NP expression. (B) RNA was extracted from sorted DCs; reverse transcription was performed and generated cDNA used in Real Time PCR reaction to determine the copy number of LCMV GP, relative to known concentration of control plasmid. ND indicates below detection limits (C) 4×10^4^ sorted DCs were cultured for 2 days in cRPMI. IL-10 levels were measured in culture supernatants of Cl13 infected (NP^pos^), exposed (NP^neg^) DCs and Naïve CD8α^neg^ DCs. n = 4–5 mice per treatment per experiment. RealTime and ELISA data from one representative of three independent experiments is shown. Standard deviation is shown.

Having identified the cell types expressing IL-10 in response to Cl13 infection *in vivo*, we examined the relationship between LCMV infection and IL-10 expression. Given that LCMV infects a variety of cell types, we tested whether the splenocytes expressing IL-10 were directly infected with the virus (Fig. 2A,B) [24]. Cell surface expression of the viral nucleoprotein (NP) was used to both identify infected cells and live cell sort directly infected DCs (NP^pos^) versus DCs exposed to virus *in vivo* but not directly infected (NP^neg^) DCs. NP expression on the surface of LCMV infected cells has been previously reported [25]. Cell surface staining for the LCMV nucleoprotein (NP) revealed that while more than 7% of cells were NP^pos^, less than 0.5% of total splenocytes expressed both IL-10 and NP, and the vast majority of IL-10^pos^ splenocytes were not directly infected (Fig. 2B). In contrast to total splenocytes, DCs expressing the viral NP on the cell surface expressed 5-fold more IL-10 than their NP^neg^ counterparts in Cl13-infected mice, demonstrating that virally infected (NP^pos^) but not merely exposed (NP^neg^) DCs express elevated IL-10 (Fig. 3C). The high IL-10 expression in uninfected splenocytes overall can likely be attributed to induction of IL-10 in naïve responder cells, as described later in this report.

### LCMV Cl13 efficiently infects CD8α^neg^ DCs

Given the ability of CD8α^pos^ DCs to prime CD8 T cell responses, and the previously reported loss of these cells during Cl13 infection, we hypothesized that the CD8α^pos^ subset might be more susceptible to Cl13 infection [5], [26]. However, we found that while Cl13 was able to infect CD8α^pos^ DC with greater efficiency than the Arm strain, the vast majority of Cl13-infected DCs were CD8α^neg^ in both C57BL/6 (Fig. 3A) and BALB/c mice (data not shown). A recent publication by the Oldstone laboratory also confirmed the finding that CD8α^neg^ DCs are the primary subset targeted in Cl13 infection [22]. The infected state of DCs as identified by LCMV NP surface staining was further confirmed with intracellular NP staining (Fig. 3A bottom panels). Additionally, quantitative Real Time PCR examining the mRNA copy number of the LCMV glycoprotein (LCMV GP per μg of RNA), revealed 45,260 copies of the viral GP in NP^pos^ DCs *versus* 1,474 (closer to the lower limit of detection) in NP^neg^ CD8α^neg^ DCs (based on surface NP staining) (Fig. 3B). A striking characteristic of virally infected (NP^pos^) DCs from Cl13 infected mice was their enhanced expression of IL-10 compared to virally exposed (NP^neg^) CD8α^neg^ DCs (Fig. 3C).

**Figure 4:**
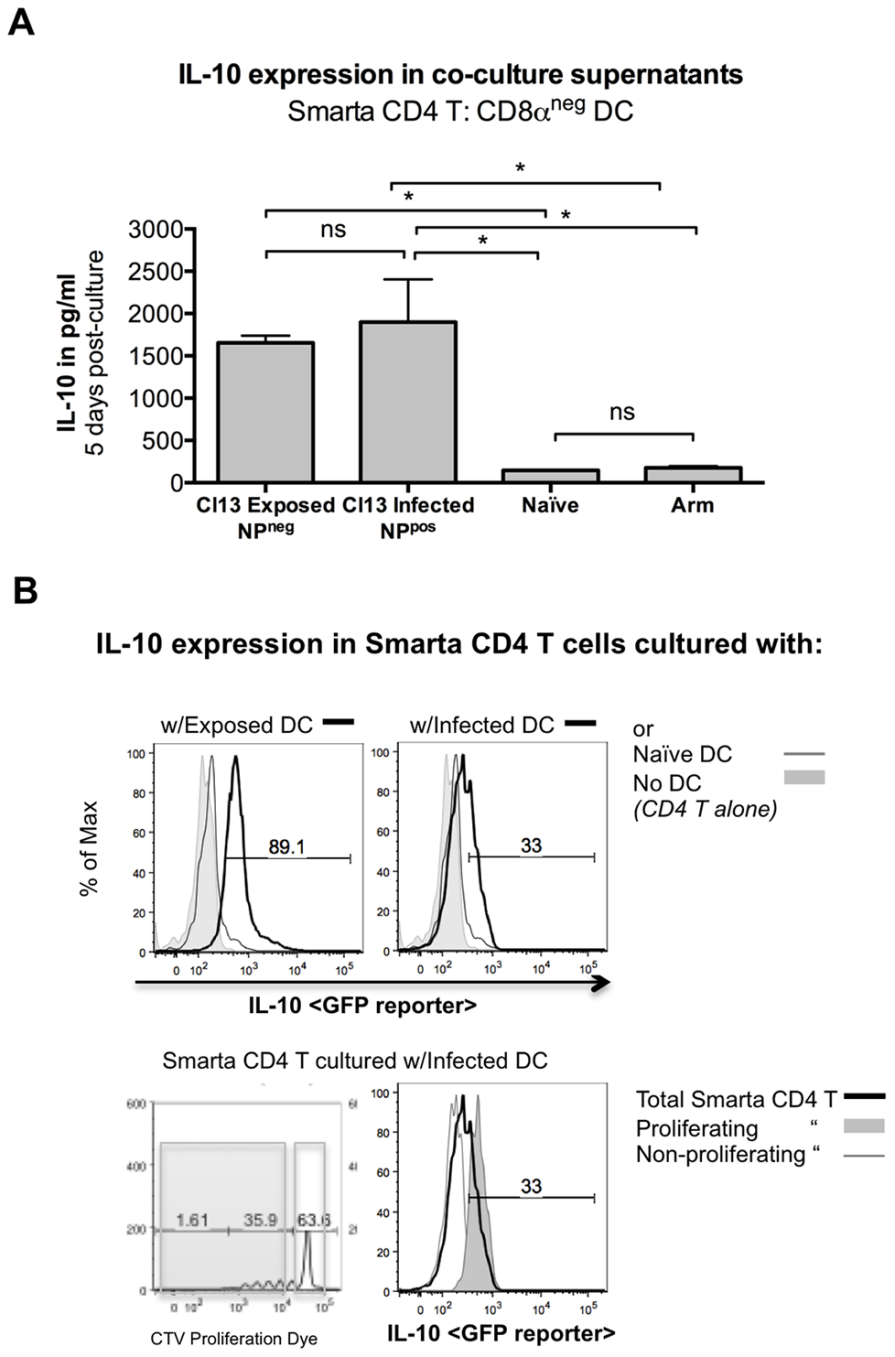
DCs from Cl13 infected mice induce IL-10 expression in naïve LCMV specific CD4 T cells. Adult C57BL/6 were infected with Cl13 or Arm and naïve spleens harvested 7 days post-infection. Splenic DCs were sorted based on infected state (NP^pos^ infected, NP^neg^ exposed) and CD8α expression (CD8α^neg^ used exclusively). (A) Sorted DCs were cultured for 5 days with LCMV TCR transgenic (Smarta) CD4 T cells and IL-10 in culture supernatants was measured by ELISA. (B) Smarta mice on an IL-10 GFP reporter background were co-cultured with sorted DCs and IL-10 expression measured by flow cytometry in gated CD4 T cells 3.5 days post co-culture. The top panels show Smarta CD4 T cultured with Cl13 exposed (NP^neg^) or infected (NP^pos^) DC (thick black line), or naïve DC (thin grey line) or Smarta CD4 T cells cultured alone (grey filled histogram). The bottom panels show Smarta CD4 T cells cultured with Cl13 infected (NP^pos^) DC. Thick black line is gated on total live Smarta CD4 T cells, thin grey line is gated on non-proliferating Smarta CD4 T cells, grey filled histogram depicts proliferating Smarta CD4 T cells (based on dilution of CellTrace^TM^Violet proliferation dye, representative CTV gating shown).

**Figure 5:**
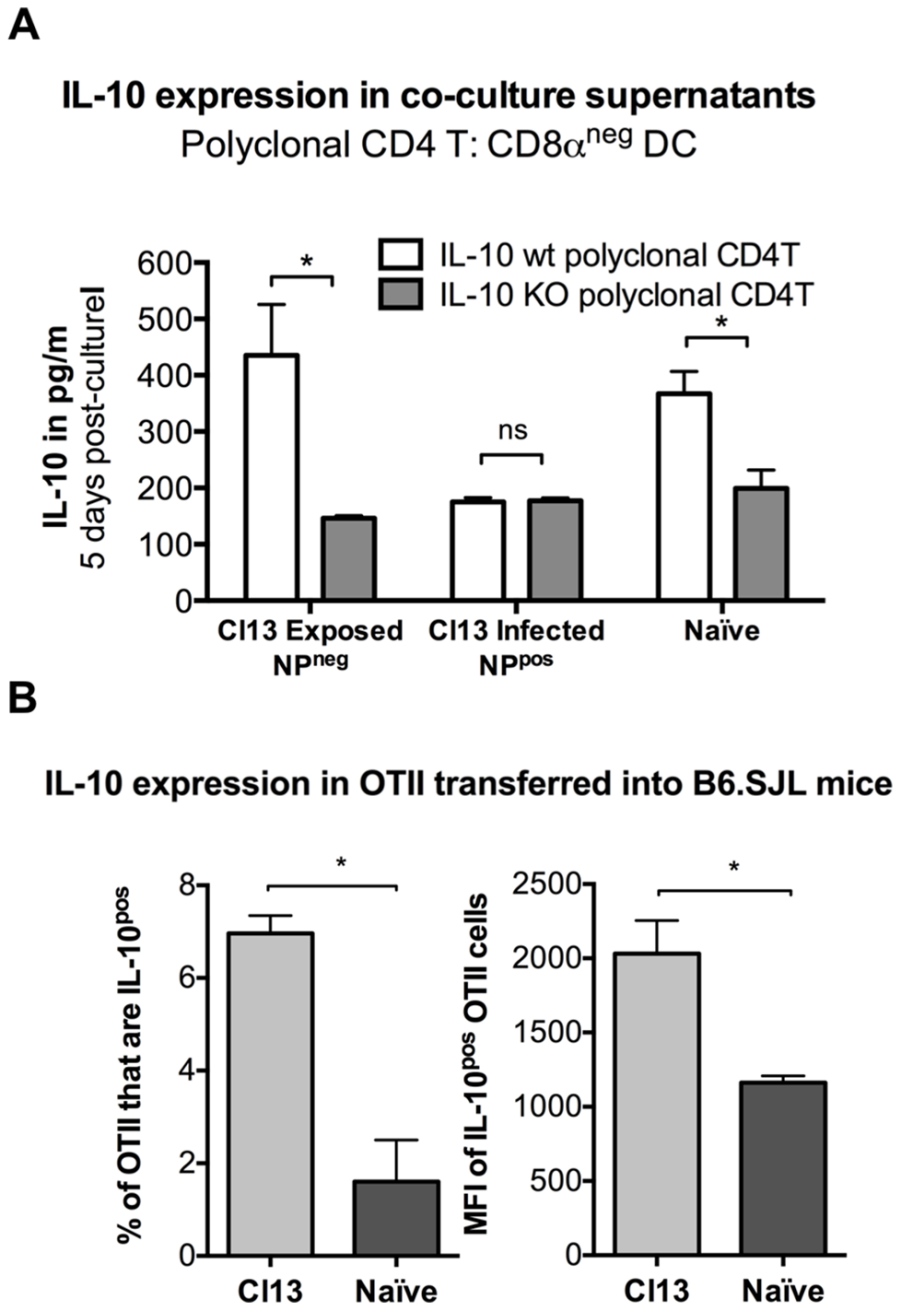
Cl13 exposed DCs induce IL-10 expression in naïve polyclonal CD4T cells. Adult C57BL/6 females were infected with Cl13 or Arm and naïve spleens harvested 7 days post-infection. Splenic DCs were sorted based on infected state (NP^pos^ infected, NP^neg^ exposed) and CD8α expression (CD8α^neg^ used exclusively). (A) 1×10^4^ sorted DCs were cultured with polyclonal CD4T cells from wildtype or IL-10 knock out C57BL/6 mice. Standard deviation is shown. (D) Polyclonal CD4T cells were harvested from OTII transgenic mice and transferred into either naïve B6.SJL or B6.SJL infected with Cl13 four days prior to T cell transfer. Spleens were harvested 3 days post-transfer (7 days post infection), stimulated in vitro with OVA peptide and analyzed by ICCS for IL-10 expression. Shown is data from 2 experiments with n = 3 Cl13 and n = 4 for naïve. Standard error of the mean is shown.

**Figure 6:**
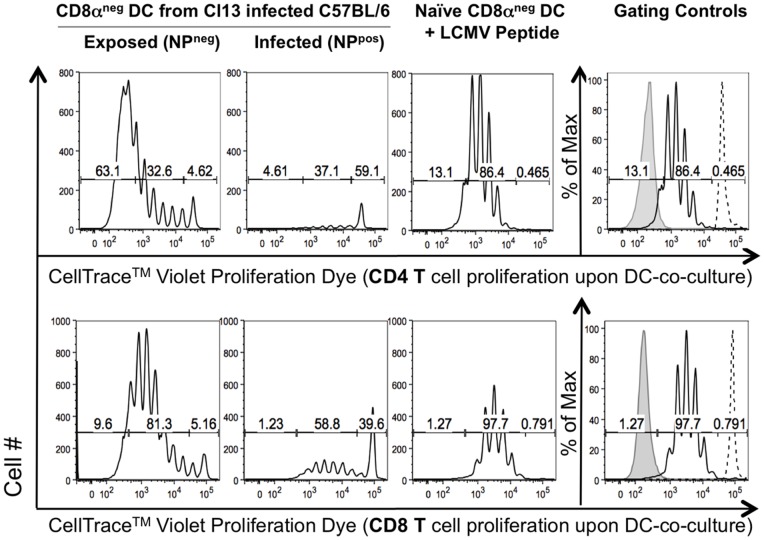
Directly infected CD8α^neg^ DCs are unable to stimulate LCMV-specific CD4 and CD8 T cell proliferation. DCs isolated from C57BL/6 mice infected *in vivo* with Cl13 7 days prior were sorted based on CD8α and LCMV NP surface expression. Sorted DCs were placed in culture with TCR transgenic LCMV specific CD4 T (Smarta) or CD8 T (P14) cells labeled with CellTrace^TM^ Violet proliferation dye (CTV) and cultured for 4.5 days. Control cultures contained LCMV peptide (GP33 and GP61) pulsed CD8α^neg^ DCs from naïve mice. Shown in the black histogram is the relative proliferation of the co-cultured Teff cells (gated on viability, CD4/8 T cell and congenic marker expression). Dashed line histograms are undivided CTV labeled Teff cells cultured alone, filled grey histograms are unlabeled cells. Each condition was set up in triplicate. Representative data from one of three independent experiments are shown.

The lack of NP at the cell surface and nearly undetectable viral RNA in NP^neg^ sorted DC, suggest that the cells are either uninfected or abortively infected at an earlier stage in the viral replication cycle than NP^pos^ DCs. Whether the NP^neg^ DCs were abortively infected prior to viral genome replication or NP production, there are clear differences in the ability of these virally exposed (NP^neg^) DCs to produce IL-10 and to prime T cell responses (Figs. 3, 4, 5, 6). These differences in functionality of virally exposed (NP^neg^) versus infected (NP^pos^) DCs are the focus of the current study.

### Infected and *in vivo* exposed CD8α^neg^ DCs induce IL-10 expression in naïve CD4 T cells

Our data thus far suggested that uninfected CD4 T cells comprised the largest population of cells expressing IL-10. While infected DCs upregulated IL-10 (Fig. 3C), both the level of expression and the number of cells expressing IL-10 was relatively low. Therefore, we investigated the possibility that enhanced tropism of Cl13 for DCs might mediate the increase in IL-10 by inducing its expression in naïve CD4 T cells. To test this possibility, co-cultures were set up with virally infected, exposed or naïve CD8α^neg^ DCs (NP^pos^ or NP^neg^ cells isolated from Cl13- or Arm- infected mice) and either polyclonal or LCMV-specific TCR transgenic, CD4 T cells (“Smarta”) [27]. We found that after 5 days, CD4 T cells cultured alone did not produce significant amounts of IL-10 (data not shown). Likewise, when DCs were cultured alone at the same number of DCs/well as in the co-cultures, IL-10 was at or below the assay’s lower limit of detection (data not shown). However, CD8α^neg^ DCs from Cl13-infected mice induced IL-10 expression in LCMV-specific CD4 T cells well above levels induced by DCs from Arm-infected or naïve mice (Fig. 4A,B).

Interestingly, in Smarta co-cultures, both Cl13-exposed and -infected CD8α^neg^ DCs were able to stimulate large quantities of IL-10. To determine whether the CD4 T cells themselves were producing IL-10, we set up parallel cultures with CD4 T cells isolated from Smarta mice on an IL-10-GFP (Smarta/GFP) reporter background and examined IL-10 (GFP) expression in the LCMV-specific CD4 T cells (Fig. 4B). When examining the total Smarta CD4 T cell population in co-cultures with exposed DCs, 89% of the CD4 T cells were GFP/IL-10+ while only 33% of total CD4 T cells cultured with infected DCs were GFP/IL-10+ (Fig. 4B). Further examination of the Smarta CD4 T/infected DC co-cultures revealed that IL-10 expression was restricted to those T cells that were proliferating, and not observed in the non-proliferating T cells (Fig. 4B). These results indicated that the LCMV-specific CD4 T cells themselves produced IL-10.

We next explored whether exposed or infected CD8α^neg^ DCs could induce IL-10 production in non-LCMV specific polyclonal CD4 T cells. Co-cultures were set up with CD8α^neg^ DCs and polyclonal CD4 T cells isolated from either wildtype (wt) or IL-10-knockout (IL-10 KO) mice on a C57BL/6 background. After 5 days in culture, the largest amount of IL-10 produced came from the Cl13-exposed (NP^neg^) CD8α^neg^ DC cultures with wt CD4 T cells, at 435 ng/ml, *versus* 102ng/ml with IL-10 KO CD4 T cells (Fig. 5A). These data more clearly demonstrated that in polyclonal CD4 T cell cultures with virally exposed DCs, both cell types contributed to total IL-10, with the majority of IL-10 originating from the CD4 T cells. On the other hand, in co-cultures of polyclonal CD4 T cells with Cl13-infected (NP^pos^) CD8α^neg^ DCs, comparable levels of IL-10 were produced when DCs were cultured with either wt or IL-10 KO CD4 T cells (Fig. 5A). These results indicated that IL-10 was derived solely from the Cl13-infected CD8α^neg^ DCs. In contrast to Smarta CD4 T cell co-cultures, where naïve DCs did not stimulate IL-10 production, in polyclonal CD4 T cell co-cultures, naïve DCs induced similar levels of IL-10 as in virally exposed DC co-cultures.

To determine whether Cl13 might be able to induce IL-10 production in polyclonal CD4 T cells *in vivo*, OTII cells (which express a transgenic TCR recognizing OVA peptide in the context of MHC class II) were adoptively transferred into congenic C57BL/6.SJL mice 4 days post infection with Cl13 (Fig. 5B). Total splenocytes were then harvested on day 7 after infection (day 3 post-transfer), and IL-10 production by donor OTII and recipient T cells (distinguished based on CD90, CD4, CD8 and CD45.1/2 expression) was measured. 6.3% of OTII cells transferred into Cl13 infected mice expressed IL-10, as compared to 1.5% of OTII cells transferred into naïve mice. Additionally, OTII cells transferred into Cl13 infected mice had a 2-fold increase in the amount of IL-10 detected (as measured by mean fluorescence intensity of the reporter) as compared to the IL10+ OTII transferred into naïve mice. These data suggest that non-LCMV specific CD4 T cells were induced to express IL-10 *in vivo*.

In summary, infected CD8α^neg^ DCs from Cl13-infected mice not only induced IL-10 production in LCMV-specific CD4 T cells, but also produced IL-10 themselves when cultured with polyclonal CD4 T cells. Virally exposed CD8α^neg^ DCs from Cl13-infected animals induced IL-10 expression in both polyclonal and virus-specific CD4 T cells.

### Directly infected (but not exposed) CD8α^neg^ DCs are unable to stimulate LCMV-specific CD4 and CD8 T cell proliferation

Recent advancements in our understanding of antigen presentation pathways have revealed multiple scenarios whereby exogenously derived antigens are presented in the context of major histocompatibility complex (MHC) I [28]–[30] (*and reviewed in* [31]–[33]). Furthermore, several studies have confirmed presentation of exogenously derived LCMV proteins by MHC I [34]–[37]. The capacity to present extracellular peptides by MHC I is particularly critical in the context of viral infection of dendritic cells, where antigen presentation pathways have been documented to be blocked by a multitude of mechanisms and the ability to effectively stimulate a CTL response hinges on presentation by uninfected DCs (reviewed in [38]–[40]). Therefore, we next asked whether direct viral infection (as compared to exposure) would impact the ability of CD8α^neg^ DCs to induce the proliferation of LCMV-specific CD4 or CD8 T cells. NP^pos^ or NP^neg^ DCs were used to stimulate LCMV-specific CD4 (Smarta) or CD8 T cells (P14, TCR transgenic cells recognizing the LCMV GP33 peptide) (Fig. 6) [41]. Cellular proliferation was measured by flow cytometry analysis of fluorescent dye dilution.

Cl13-exposed (NP^neg^) DCs were able to stimulate more than 95% of the CD4 T cells to proliferate, thus greatly expanding the numbers of CD4 T cells in the culture by day 4.5. While in infected DC cultures nearly 42% of the CD4 T cells had proliferated by day 4.5, significantly lower numbers of CD4 T cells were found in cultures with virally infected as compared to exposed DCs. When total cell number and degree of proliferation were taken into account, infected DCs were unable to effectively stimulate CD4 or CD8 T cell proliferation, whereas NP^neg^ exposed DCs were able to efficiently stimulate T cell proliferation.

Analysis of cell surface expression of MHC I, MHC II, CD80 and CD86 revealed no significant difference between Cl13-exposed and Cl13-infected CD8α^neg^ DCs relative to isotype control (data not shown). Given that proteins critical to antigen presentation and co-stimulation were present in both infected and exposed DCs, we hypothesized that infected DCs may be defective in their ability to process viral antigen. We found that the addition of LCMV peptides GP33 and GP61 to the infected CD8α^neg^ DC: T cell co-cultures was able to restore proliferation of LCMV-specific CD8 and CD4 T cells (Fig. S1). However the total cell number of T cells in the infected CD8α^neg^ DC co-cultures was still reduced relative to exposed CD8α^neg^ DC, suggesting that additional suppressive factors (such as elevated IL-10 seen in Fig. 3C) limit the extent of proliferation.

## Discussion

**Figure 7:**
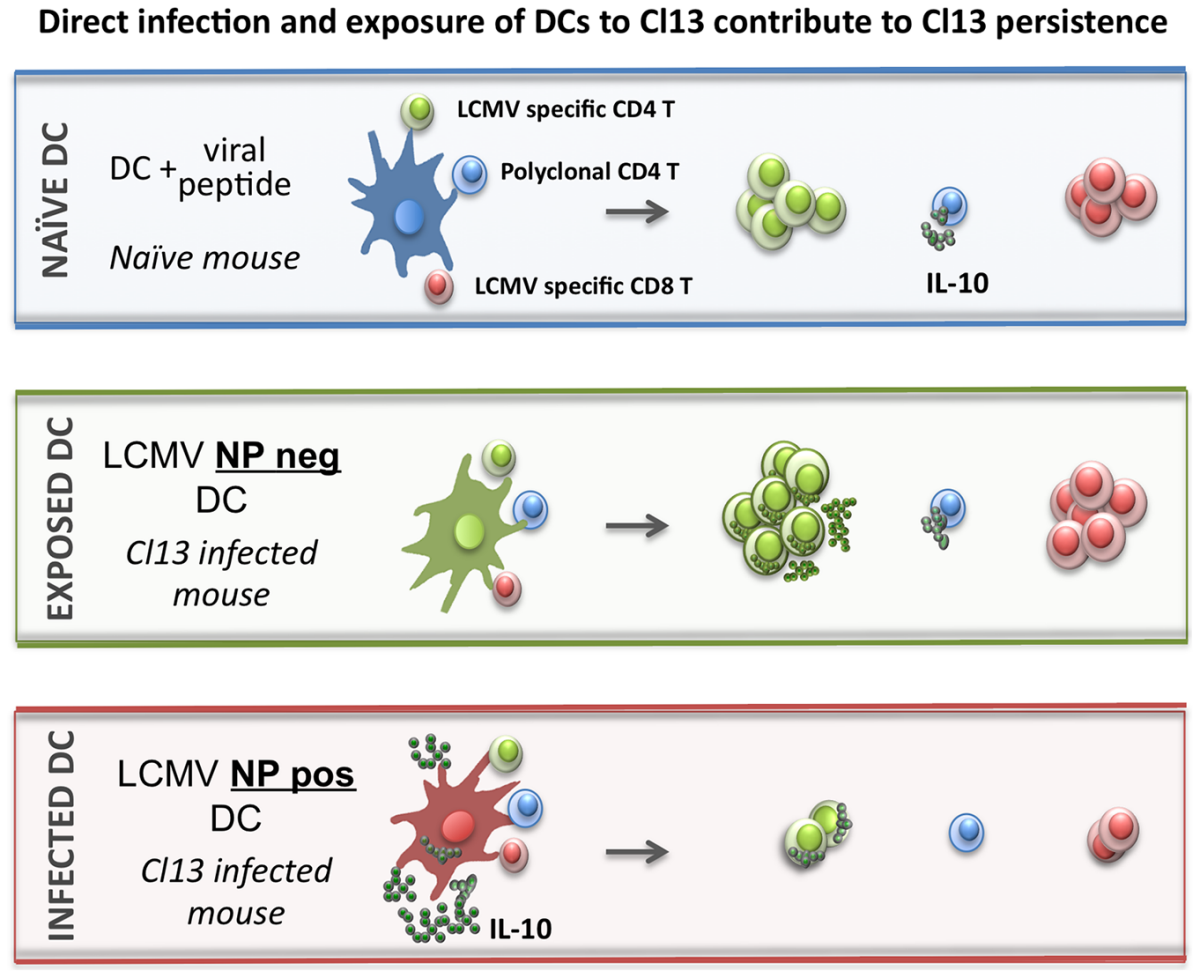
Direct infection and exposure of CD8α^neg^ DCs to LCMV Cl13 differentially contribute to Cl13 persistence. Naïve DCs, those harvested from uninfected mice, incubated with viral peptide are able to stimulate both LCMV specific CD4 and CD8 T cell proliferation, as well as induce IL-10 expression in naïve polyclonal CD4 T cells. Virally exposed DCs, those isolated from LCMV Cl13 infected mice and negative for surface expression of viral NP, are able to stimulate both LCMV specific CD4 and CD8T cell proliferation and also induce IL-10 expression in naïve polyclonal CD4 T cells. Smarta CD4 T cells cultured with Cl13 exposed DCs produce copious amounts of IL-10. Cl13 infected DC, positive for surface expression of viral NP, express enhanced levels of IL-10 as compared to either naïve or exposed DC, are unable to stimulate LCMV specific CD4 or CD8 T cells. The few CD4 T that are induced to proliferate in the presence of infected DCs express elevated levels of IL-10 as compared to either those cultured alone or in the presence of naïve DCs and LCMV peptide.

In this study we set out to determine which cell types would produce IL-10 in response to Cl13 infection *in vivo*, and what role enhanced viral tropism for DCs may play in the induction and maintenance of elevated IL-10 levels during Cl13 infection. We report that directly infected, in contrast to virally exposed, CD8α^neg^ DCs from Cl13-infected mice express elevated IL-10 and suppress CD4 and CD8 T cell proliferation *in vitro*. In addition, we found that DCs from Cl13-infected animals promote IL-10 production in naïve polyclonal and virus-specific CD4 T cells. While both direct infection and exposure of CD8α^neg^ DCs were found to contribute to elevated IL-10, different modes for each subset were identified. Virally exposed (NP^neg^) DCs were found to stimulate IL-10 production in both polyclonal and LCMV-specific CD4 T cells. Infected DCs, in addition to producing elevated IL-10, induced IL-10 expression in proliferating LCMV-specific CD4 T cells (summarized in Fig. 7). These data support a pivotal role for DCs in initiating and perpetuating an IL-10 production cascade contributing to T cell exhaustion and long-term establishment of LCMV Cl13 with its host.

The importance of IL-10 as a master regulator in chronic LCMV infection was first illustrated in 2006 by both the Oldstone group and our laboratory [3], [5]. Using two different model systems, one employing IL-10 KO mice, the other utilizing an anti-IL-10Rα antibody, both groups demonstrated that blockade of IL-10 signaling alone was sufficient to clear an otherwise chronic LCMV infection [3], [5]. We demonstrated that when total splenocytes are exposed to virus *in vitro*, multiple cell types respond by producing IL-10 [5]. While these findings represented important steps in our understanding of the broad range of cells capable of responding to LCMV, it still remained unknown which cell types would express IL-10 *in vivo*.

Several recent publications have illuminated different aspects of the LCMV IL-10 scenario. One such study described the presence of IL-10-expressing APCs during chronic LCMV infection [21]. Phenotypic analysis of the IL-10^pos^ APC identified primarily macrophages with elevated surface expression of MHC and co-stimulatory molecules (MHC I, II, CD80 and CD86), as well as increased inhibitory molecules PD-L1 and (to a lesser extent) PD-L2. The inhibitory effect of IL-10-expressing APCs on LCMV-specific CD4 T cell proliferation *in vitro* was completely abrogated by the addition of neutralizing antibody to IL-10. Wilson *et al.* did not distinguish between virally exposed and directly infected APCs, but rather focused on IL-10-producing macrophages and APCs in general. Their conclusion that the IL-10-expressing APCs were not infected was based upon infectious center assays, which determine the level of productive infection, but cannot identify the level of abortive infection. Our studies also showed that the vast majority of DCs were not productively infected (data not shown), but rather their function was altered in the course of an abortive viral infection as demonstrated by the presence of viral RNA (qPCR for viral GP) as well as surface and intracellular expression of viral NP protein. Abortive viral infections, characterized by the lack of progeny virus but presence of viral particles within the cell, have been documented to alter the function of DCs. Effects of viral and cellular protein present in the incoming virion, PAMP recognition, interferon induction, PKR stimulation can all modulate DC maturation and function, independent of the generation of infectious progeny virus. In terms of immune evasion, disruption of DC function is a kamikaze, albeit effective, outcome of abortive infection. In this study, we observed that abortively infected DCs (LCMV NP^pos^) expressed elevated IL-10 and were altered in function as compared to their exposed (LCMV NP^neg^) DC counterparts.

In a previous study by the Oldtsone group utilizing IL-10 conditional KO in combination with the IL-10-eGFP reporter mice VertX, it was shown that selective deletion of CD11c-specific IL-10 reduced serum IL-10 levels by ∼50% [22]. This finding led the authors to conclude that CD8α^neg^ DCs constitute the main source of IL-10, with macrophages being a minor contributor to overall IL-10 levels. These conclusions are also in agreement with our finding that DCs play a central role in priming IL-10 production in other cell types, which we found to be mainly CD4 T cells. Interestingly, in the absence of DC-specific IL-10 production, systemic IL-10 levels were decreased by 50% but not completely abrogated, suggesting the existence of other, possibly compensatory, mechanisms contributing to elevated IL-10 in the serum. In fact the authors found that deletion of CD4 T cells did not affect overall IL-10 levels, suggesting that under certain circumstances IL-10 produced by other cell types (such as DCs themselves) may contribute to increased levels in the serum. While our data do not support the conclusion that DCs *alone secrete* the majority of serum IL-10 (in animals with all cellular compartments intact), data from both our and the Oldstone groups do support a central role of infected CD8α^neg^ DCs for increased IL-10 levels in the serum.

More recently Richter and colleagues have reported that depletion of DCs or CD4 T cells resulted in significant loss of total IL-10 [42]. Our data demonstrating that DCs from Cl13 infected mice are able to induce IL-10 in CD4 T cells explains both the Richter and Ng findings, where both loss of DCs and loss of CD4 T cell derived IL-10 lead to dramatic loss of total IL-10. Our model supports the findings of these groups and builds upon our current understanding of the complex role of DCs and IL-10 in promoting chronic viral infection.

The data we present here showing both elevated IL-10 expression in infected (versus virally exposed or naïve) CD8α^neg^ DCs together with the finding that these cells are able to induce IL-10 expression in naïve CD4 T cells likely explains how infection of CD8α^neg^ DCs contributes to total IL-10. The inhibitory nature of infected CD8α^neg^ DCs was validated in functional studies demonstrating inhibition of LCMV-specific CD4 and CD8 T cell proliferation, and induction of IL-10 expression in naïve CD4 T cells.

Our finding that infected DCs are able to promote IL-10 expression in naïve CD4 T cells, coupled with the inability of infected DCs to stimulate both CD4 and CD8 T cell proliferation, introduces previously unrecognized roles for direct infection and exposure of DCs in triggering a cascade effect of IL-10 production in uninfected cells, both LCMV-specific and polyclonal. Our study provides insight into the seemingly disparate findings as to which cell types are key producers of IL-10 *in vivo*, and how infection of DCs contributes to both the IL-10 production loop and T cell exhaustion in chronic viral infection.

## Materials and Methods

### Ethics statement

All animal experimental protocols were approved by the La Jolla Institute for Allergy and Immunology Institutional Animal Care and Use Committee, (PHS assurance # A3779-01). The LIAI Department of Laboratory Animal Care complies with the *Guide for The Care and Use of Animals* and is an AAALAC (Association for Assessment and Accreditation of Laboratory Animal Care) International accredited unit.

### Mice

6-week-old BALB/c, C57BL/6, B6.SJL and OTII mice were purchased from the Jackson Laboratory and housed for two weeks prior to infection or spleen harvest. Smarta, CD4+ TCR transgenic mice specific for the GP_61–80_ LCMV peptide were a gift from Shane Crotty (La Jolla Institute for Allergy & Immunology, La Jolla, CA) [27]. P14 mice are TCR transgenic mice specific for the GP_33–41_ LCMV peptide on MHC I [41]. IL-10-GFP reporter TIGER mice were a gift from Richard Flavell (Yale School of Medicine, New Haven, CT) [23].

### Viral strains, preparation, quantification and infection

LCMV Clone 13 and Armstrong 53b strains were triple plaque purified on Vero cells and stocks prepared by passage through BHK-21 cells as previously described [5]. 8- week old female mice were infected with either 2×10^6^ PFU of LCMV Clone 13 via retro-orbital injection or 2×10^5^ PFU of LCMV Armstrong via intra-peritoneal injection.

### DC isolation and characterization

Spleens were harvested at the indicated times postinfection and single cell suspensions prepared by collagenase D digestion (Roche), followed by RBC lysis. B (CD19^+^) cells and T (CD90^+^) cells were depleted from the splenocyte preparation via positive selection (antibody clones: CD19 clone 1D3 and CD90 clone G7, BD Pharmingen) and magnetic bead separation (anti-rat IgG Dynal beads, Invitrogen). Following B and T cell depletion CD11c+ DCs were positively selected via MACS bead separation (Miltenyi). Total CD11c+ cells were counted, FcRc blocked (anti-CD19/CD32 BD Pharmingen then blocked with unconjugated anti-rat Fc FAB [Jackson Immuno Research Laboratories, Inc.]) and stained for surface expression of LCMV-NP (VL4 hybridoma supernatant, gift from M.Oldstone in combination with secondary PE-conjugated anti-rat IgG2a), then CD11c (Clone HL3, BD Pharmingen), CD90.2 (Clone 30-H12, BD Pharmingen), CD8α (Clone 53–6.7 BD Pharmingen), CD19 (Clone 1D3, BD Pharmingen). CD11c^hi^, CD90^−^, CD19^−^, CD8α^ pos/neg^ and LCMV NP^pos/neg^ DCs were then sorted by FACS (Aria, Becton Dickinson). Samples were run on an LSRII (Becton Dickinson) and analyzed with FlowJo software (Treestar). Sorted DCs (4×10^4^ cells/well) were cultured alone for 48 hours in RPMI 1640 (Invitrogen) supplemented with 5mM Hepes, 10% FBS (HyClone), 1% Penicillin Streptomycin (Life Technologies), 1% Glutamax (Life Technologies) and 50 μM β-mercaptoethanol (Life Technologies). Supernatants were collected and analyzed by ELISA for IL-10 expression.

### RNA extraction, cDNA synthesis and qPCR

Total RNA was extracted from FACS sorted DCs using RNeasy Mini kit (Qiagen) per manufacturer’s instructions. Equal amounts of total RNA were used to generate cDNA via reverse transcription with the High Capacity RNA to DNA kit (Applied Biosystems). Quantitative Real Time PCR reactions were performed as described in [43]. In brief, cDNA was amplified using LCMV glycoprotein primers (GP forward primer: CATTCACCTGGACTTTGTCAGACTC, GP reverse primer: GCAACTGCTGTGTTCCCGAAAC), Sybr Green kit (Roche) and LightCycler 480 (Roche) thermocycler. Quantification was performed using a pSG5-GP plasmid DNA standard curve.

### CD4 T cell DC co-culture

CD4 T cells were isolated from either B6 or Smarta TCR transgenic mice via negative selection by antibody depletion of B220 (Clone RA3-6B2, BD Pharmingen), CD11b (Clone M1/70, BD Pharmingen), CD16/32 (Clone 2.4G2, BD Pharmingen), IA/IE (Clone 2G9, BD Pharmingen) and CD8α (Clone 53–6.7, BD Pharmingen) with magnetic bead separation (anti-rat IgG Dynal beads, Invitrogen).

Isolated CD4 T cells were cultured either alone or with FACS purified DCs in 96-well round bottom plates for 5 days in RPMI 1640 (Invitrogen) supplemented with 5 mM Hepes, 10% FBS (HyClone), 1% Penicillin Streptomycin (Life Technologies), 1% Glutamax (Life Technologies) and 50μM β-mercaptoethanol (Life Technologies). Supernatants were collected and analyzed by ELISA for IL-10 expression.

### ELISA

Mouse IL-10 ELISA was performed on pre-cleared co-culture supernatants using the BioLegend ELISA MAX Standard anti-mouse IL-10 kit per manufacturers directions. Plates were read at 450 and 570nm (SpectraMax 250, Molecular Devices).

### CD4 and CD8 T cell proliferation assay

CD8 T cells were purified from the spleens of adult P14 mice via negative selection by antibody depletion of B220 (RA3-6B2, BD Pharmingen), CD11c (HL3, BD Pharmingen), CD11b (M1/70, BD Pharmingen), CD16/32 (2.4G2, BD Pharmingen), IA/IE (2G9, BD Pharmingen) and CD4 (L3T4 RM4-5, BD Pharmingen) with magnetic bead separation (anti-rat IgG Dynal beads, Invitrogen). CD4 T cells were purified from the spleens of adult Smarta mice via negative selection by antibody depletion of B220 (RA3-6B2, BD Pharmingen), CD11b (M1/70, BD Pharmingen), CD16/32 (2.4G2, BD Pharmingen), IA/IE (2G9, BD Pharmingen), CD8α (Clone 53–6.7, BD Pharmingen) with magnetic bead separation (anti-rat IgG Dynal beads, Invitrogen). Cells were counted and labeled with CellTrace^TM^ Violet proliferation dye (Invitrogen) according to manufacturers instructions. FACS sorted DCs were plated at 1×10^4^ cells/well of a round bottom 96 well plate together with 1×10^5^ cells/well of purified CD8 T cells or 3×10^4^ CD4 T cells in RPMI 1640 (Invitrogen) supplemented with 5 mM Hepes, 10% FBS (HyClone), 1% Penicillin Streptomycin (Life Technologies), 1% Glutamax (Life Technologies) and 50μM ≤β-mercaptoethanol (Life Technologies). As positive control for T cell proliferation, T cells were co-cultured with GP33 or GP61 (Abgent) peptide pulsed naïve CD8α^neg^ DCs.

### Statistics

Statistics were calculated using GraphPad Prism (version 6) software. Comparisons between the different groups were performed with Mann-Whitney t-tests. Data are expressed as mean ± standard error. * P≤0.05, ** P<0.005, ***P<0.0005.

## Supporting Information

Figure S1 **Addition of exogenous peptide restores infected DC stimulation of T cell proliferation.** DCs isolated from C57BL/6 mice infected *in vivo* with Cl13 7 days prior were sorted based on CD8α and LCMV NP surface expression. Sorted DCs were placed in culture with TCR transgenic LCMV specific CD4 T (top panels) or CD8 T cells (bottom panels) labeled with CTV proliferation dye and cultured for 4.5 days with or without LCMV peptide (GP33 and GP61) as indicated. Control cultures contained CD8α^neg^ DCs from naïve mice with peptide and are shown in the right side panels with CTV stained T cells alone and unstained splenocytes as indicated. Representative data from one of three independent experiments is shown.
(TIF)
