# Supplementary figures and images for: Direct Infection of Dendritic Cells during Chronic Viral Infection Suppresses Antiviral T Cell Proliferation and Induces IL-10 Expression in CD4 T Cells
Source: PLoS One. 2014 Mar 10;9(3):e90855. doi: 10.1371/journal.pone.0090855 (PMC3948950; doi:10.1371/journal.pone.0090855)

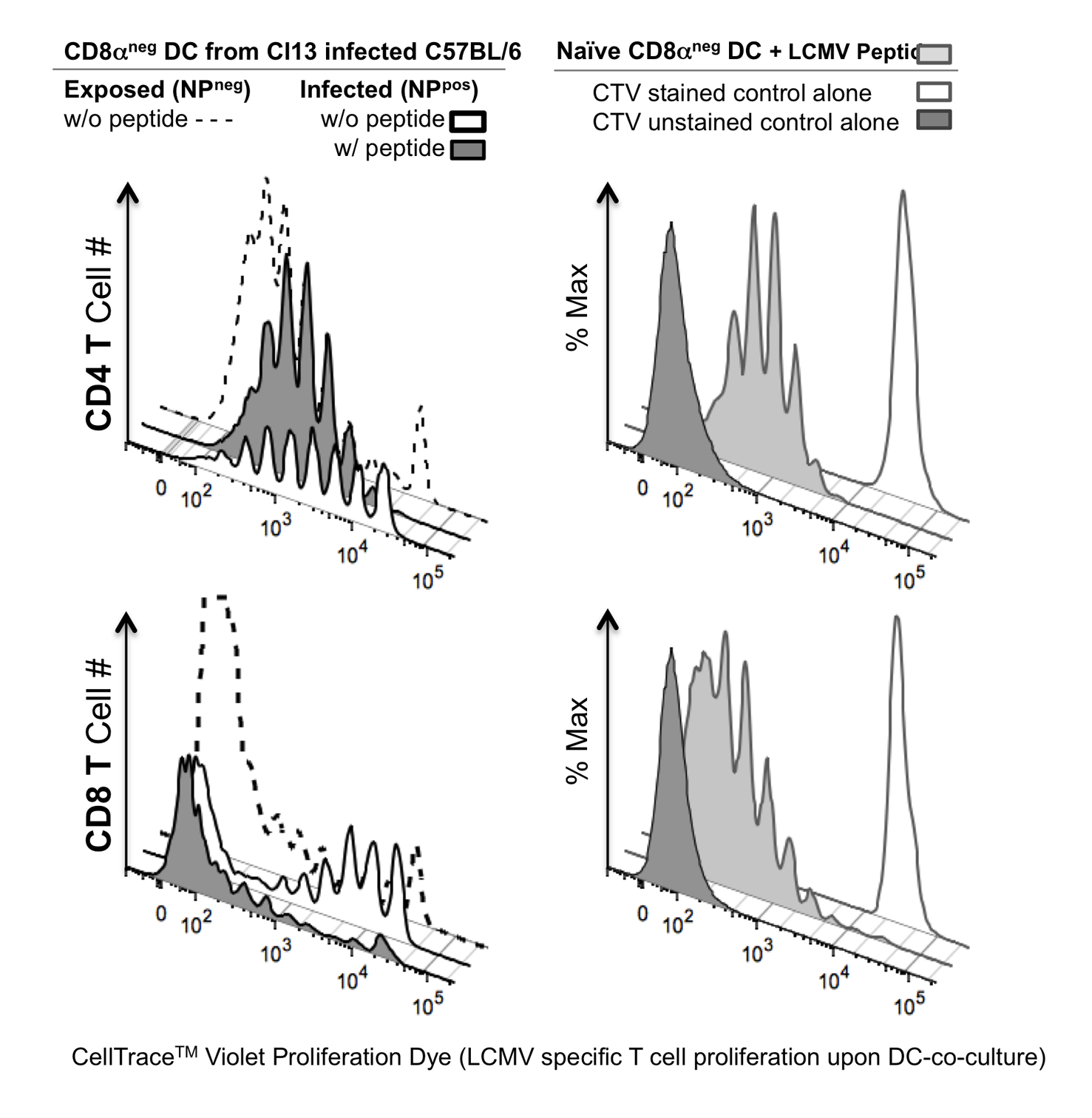

Supplement: Figure S1 — Addition of exogenous peptide restores infected DC stimulation of T cell proliferation. DCs isolated from C57BL/6 mice infected in vivo with Cl13 7 days prior were sorted based on CD8α and LCMV NP surface expression. Sorted DCs were placed in culture with TCR transgenic LCMV specific CD4 T (top panels) or CD8 T cells (bottom panels) labeled with CTV proliferation dye and cultured for 4.5 days with or without LCMV peptide (GP33 and GP61) as indicated. Control cultures contained CD8αneg DCs from naïve mice with peptide and are shown in the right side panels with CTV stained T cells alone and unstained splenocytes as indicated. Representative data from one of three independent experiments is shown. (TIF) [file pone.0090855.s001.tif]
